# Autoimmune and Neoplastic Thyroid Diseases Associated with Hepatitis C Chronic Infection

**DOI:** 10.1155/2014/935131

**Published:** 2014-10-13

**Authors:** Poupak Fallahi, Silvia Martina Ferrari, Ugo Politti, Dilia Giuggioli, Clodoveo Ferri, Alessandro Antonelli

**Affiliations:** ^1^Department of Clinical and Experimental Medicine, University of Pisa, Via Savi 10, 56126 Pisa, Italy; ^2^Department of Medical, Surgical, Maternal, Pediatric and Adult Sciences, University of Modena and Reggio Emilia, Via del Pozzo 71, 41100 Modena, Italy

## Abstract

Frequently, patients with hepatitis C virus (HCV) chronic infection have high levels of serum anti-thyroperoxidase and/or anti-thyroglobulin autoantibodies, ultrasonographic signs of chronic autoimmune thyroiditis, and subclinical hypothyroidism, in female gender versus healthy controls, or hepatitis B virus infected patients. In patients with “HCV-associated mixed cryoglobulinemia” (MC + HCV), a higher prevalence of thyroid autoimmune disorders was shown not only compared to controls, but also versus HCV patients without cryoglobulinemia. Patients with MC + HCV or HCV chronic infection show a higher prevalence of papillary thyroid cancer than controls, in particular in patients with autoimmune thyroiditis. Patients with HCV chronic infection, or with MC + HCV, in presence of autoimmune thyroiditis, show higher serum levels of T-helper (Th)1 (C-X-C motif) ligand 10 (CXCL10) chemokine, but normal levels of Th2 (C-C motif) ligand 2 chemokine, than patients without thyroiditis. HCV thyroid infection could act by upregulating CXCL10 gene expression and secretion in thyrocytes recruiting Th1 lymphocytes that secrete interferon-*γ* and tumor necrosis factor-*α*. These cytokines might induce a further CXCL10 secretion by thyrocytes, thus perpetuating the immune cascade, which may lead to the appearance of autoimmune thyroid disorders in genetically predisposed subjects. A careful monitoring of thyroid function, particularly where nodules occur, is recommended in HCV patients.

## 1. Introduction

About 130–170 million people worldwide have been infected by hepatitis C virus (HCV) [1]. Hepatocytes represent the major site of viral replication, and the replication of HCV is present in extrahepatic tissues and peripheral blood mononuclear cells [2].

Previous studies have shown that 38–76% of patients with chronic HCV infection develop at least one extrahepatic manifestation (EHM) [3, 4].

An association between HCV and mixed cryoglobulinemia (MC) was first described; subsequently, the involvement of many organs and systems was reported (kidney, skin, eyes, joints, and nervous system). The infected extrahepatic tissues might act as a reservoir for HCV [5] and play a role in both HCV persistence and reactivation of infection. HCV, as an etiological agent replicating and expressing viral proteins in extrahepatic tissues itself, contributes to EHM associated with chronic HCV infection. An important feature of HCV is that the virus avoids immune elimination; a consequence is chronic infection and an accumulation of circulating immunocomplexes and autoimmune phenomena [6–8], as recently Cheng et al. have demonstrated in their study among 297 Chinese patients [9].

These EHM mainly include autoimmune disorders [10–12] such as MC [13, 14] and Sjogren's syndrome and endocrinological diseases as autoimmune thyroid disorders (AITD) and type 2 diabetes [15–17].

## 2. Autoimmune Thyroiditis

Hashimoto's thyroiditis or autoimmune chronic thyroiditis (AT) is among the most common thyroid diseases. AT is the most widespread thyroiditis form and its prevalence is definitely more frequent in female gender and in the elderly. The incidence in female gender is of 3,5 cases/1000 subjects per year, while in men it is lower (0,8 cases/1000 persons per year): there is a remarkable variability in different geographic areas [18].

AT is an organ-specific autoimmune disease, morphologically characterized by a chronic lymphocytes infiltration of thyroid and the presence of circulating autoantibodies such as antiperoxidase (AbTPO) and antithyroglobulin (AbTg). The inflammatory process leads to a follicular destruction; indeed, AT is the most common cause of hypothyroidism in areas of iodine sufficiency [19].

Occasionally, thyroid stimulating hormone (TSH) receptor blocking antibodies can be responsible of an atrophic form of AT; more rarely, anti-TSH receptor stimulating antibodies can cause a transient form of hyperthyroidism (hashitoxicosis) [20].

Risk factors associated with AT are numerous [21–24].

- Age: the prevalence of disease tends to increase with age.
- Genetic: a significant association between Hashimoto's thyroiditis and some histocompatibility antigens (HLA-DR, HLA-DR5, and some DQ alleles) is demonstrated. Many other susceptibility genes have been associated with AT; for example, specific CTLA4 gene polymorphisms are linked to a possible development of antithyroid antibodies [25].
- Iodine: an increased AT prevalence is observed in areas of iodine sufficiency, compared with iodine-deficient areas [26, 27].
- Selenium: a selenium deficit is linked to a higher AT prevalence [28].
- Irradiation: AT occurs more frequently after the exposure to low doses of radiations [26].
- Cytokine: the treatment with Interferon- (IFN-) *α*, or with Interleukin- (IL-) 2, can promote the onset of AT in predisposed patients [29].
- Infections: it was seen that several viral infections can predispose to an AT in animals. Moreover, different studies tried to associate AT with viral infections in humans with conflicting results [30–33].

## 3. Chronic HCV (CHC) Infection and Thyroid

### 3.1. CHC Infection and Thyroid Autoimmunity

In a first study, Tran et al. report two cases of Hashimoto's thyroiditis associated with chronic active HCV infection, suggesting that HCV infection might be involved in the appearance of AT [34, 35].

The prevalence of HCV infection in patients with different thyroid disorders has been evaluated by several studies with conflicting results. Duclos-Vallée et al. evaluated the prevalence of HCV infection in 200 patients with thyroid diseases; among 50 patients with simple goiter, none were anti-HCV-positive; among 50 individuals with goiter, 2 were positive; among 5 individuals with myxedema, 2 were positive; among 50 patients with Hashimoto's thyroiditis, 12 were positive. These results suggested that HCV infection might be associated with AT [36]. Recently, Yang et al. compared 462 persons with positive AbTPO and/or AbTg to 360 persons with antibody negativity and no difference in the prevalence of anti-HCV positivity between the 2 groups (1.3% versus 0.53%; *P* > 0.05) was found [37]. In a study conducted by Marconcini et al., 66 HCV+ patients were evaluated and AbTPOs were detected in 4/54 (7.4%) of the patients, whereas AbTgs were detected in none of the patients (0/48) [38].

Conflicting results have been reported from earlier studies of patients with CHC, with some supporting an association of HCV infection with AITD [39–47] and others not [48, 49].

However, some of the earlier studies were negative because of the lack of control for factors which may affect the development of thyroid autoimmunity, such as iodine intake [50].

Indeed, the largest study about HCV and thyroiditis, in which iodine deficiency was evaluated, demonstrated that both hypothyroidism and thyroid autoimmunity were significantly more common in patients with HCV compared to controls [41].

The prevalence of thyroid disorders in 630 consecutive patients with chronic hepatitis due to HCV infection was investigated; all patients were free of cirrhosis and hepatocarcinoma and were not on interferon treatment. Three control groups were included: (a) 389 subjects from an iodine-deficient area, (b) 268 persons living in an area of iodine sufficiency, and (c) 86 patients > 40 years of age with chronic hepatitis B. Levels of thyroid-stimulating hormone (TSH), free T4 (FT4), and free T3 (FT3), as well as AbTgs and AbTPOs, were measured. Mean TSH levels were higher (*P* = 0.001) and FT3 and FT4 levels were lower (*P* < 0.0001) in patients with CHC than in all other groups. Patients with CHC were more likely to have hypothyroidism (13% (*n* = 82)), AbTgs (17% (*n* = 108)), and AbTPOs (21% (*n* = 132)) than were any of the other groups. The results of this study suggested that both hypothyroidism and thyroid autoimmunity are more common in patients with CHC, even in the absence of cirrhosis, hepatocellular carcinoma, or interferon treatment, than in HCV-negative controls or in patients with chronic hepatitis B infection [41].

Evidence for this association also came from a study that reported a higher prevalence of hypothyroidism and AbTgs in untreated children with CHC compared to healthy non-HCV infected controls [51]. In most studies, examining the frequency of thyroid disorders in patients with HCV, approximately 10–15% of the patients had positive thyroid antibodies before the beginning of the therapy with IFN [52–58]. Moreover, pooling of data from controlled studies on HCV infection and thyroid autoimmunity demonstrated a significant increase in the risk of thyroiditis in HCV patients [59]. A large study which included 146394 patients infected with HCV confirmed these results showing a significant increased risk for thyroiditis [60]. This was a retrospective cohort study of users of US Veterans Affairs health care facilities from 1997 to 2004, which included 146394 CHC patients who had at least 2 visits and 572293 patients uninfected with HCV. The thyroiditis risk was significantly increased in HCV patients. Since 97% of HCV patients were men and it is well known that male gender has a lower risk of thyroiditis than female, this result is particularly interesting [60].

The presence of higher risk of AT in female gender increased circulating levels of AbTPOs and increased risk of hypothyroidism in female gender and AbTPO-positive subjects characterized the pattern of thyroid disorders observed in HCV infection [59, 61, 62].

Despite their remarkable therapeutic efficacy, IFN-*α* adverse effects are well-known, from influenza-like symptoms to hematologic effects, neuropsychiatric symptoms, and thyroid diseases [63]. In particular, previous studies showed that female gender is one of the most common risk factors that predict the development of AITD during interferon therapy [64, 65]. An association between IFN-*α* and thyroid disease was recognized as early as 1985 in patients who have been treated with IFN-*α* for breast cancer [66]. Later, several cases have reported the possible association between thyroid disease and IFN-*α* [67]. Different forms of IFN induced thyroid autoimmunity have been identified, such as GD, thyroiditis, and subclinical hypothyroidism [68]. Graves' hyperthyroidism is the less common type, because only 20–25% of all patients with IFN-related thyrotoxicosis are linked to Graves' disease (GD) induced by circulating thyroid receptor antibodies (TRAb) [69, 70].

Interferon induced thyroiditis (IIT) can be divided into two main groups: autoimmune type and nonautoimmune type [71]. The former can manifest as HT and GD and sometimes may be related to the production of thyroid autoantibodies without clinical disease.

Another interesting classification of interferon induced hyperthyroidism has been proposed by Czarnywojtek et al. [72] (in comparison with amiodarone induced thyrotoxicosis (AIT)): (I) type 1, corresponding to type I amiodarone induced thyrotoxicosis (AIT): (a) GT without TAO and (b) GT with TAO (mild or severe); (II) type 2 destructive thyrotoxicosis, partially analogous to type II AIT: (a) asymptomatic: silent thyroiditis and (b) symptomatic; and (III) type 3 unknown aetiology, partial analogy to type III AIT—undefined or mixed.

The presence of thyroid autoantibodies before the initiation of IFN-*α* therapy is an important risk factor for the development of IIT. In HCV-positive individuals, the progression toward hypothyroidism, in thyroid autoantibodies positive patients who undergo IFN-*α* treatment, is often associated with an increase in antibody titers [73]. Furthermore, Prummel and Laurberg showed that positive pretreatment AbTPOs are an important risk factor for the development of thyroid dysfunction [74]. There is also an obvious link between female sex, old age, and genetic predisposition with the development of antibodies [75, 76].

### 3.2. Cryoglobulinemia and Thyroid Diseases

Few anecdotal studies evaluated AITD in patients with cryoglobulinemia [77, 78].

A case-control prospective study has been conducted to evaluate thyroid disorders in 93 MC + HCV patients, matched by sex and age (±2 years), to 93 patients with CHC without MC and 93 healthy (HCV-negative) controls from the local population [79, 80]. The following thyroid autoimmune abnormalities were significantly more frequent in MC + HCV patients than in HCV-negative controls: serum AbTPO levels (28% versus 9%), serum AbTPO and/or AbTg levels (31% versus 12%), AT (35% versus 16%), and subclinical hypothyroidism (11% versus 2%). Serum AbTPOs were also significantly more frequent in MC + HCV patients than in CHC controls (28% versus 14%). A higher prevalence of thyroid disorders in patients with MC + HCV not only with respect to controls, but also with respect to HCV patients without cryoglobulinemia was shown, suggesting a careful monitoring of thyroid function in these patients [80].

The presence of a higher risk of AT and hypothyroidism and increased circulating levels of AbTPO, in female gender, characterized the pattern of thyroid disorders observed in MC + HCV infection, similarly to HCV patients without MC [59, 61].

### 3.3. CHC Infection and Thyroid Cancer

A high prevalence of papillary thyroid cancer (PTC) was first observed in 139 HCV patients (2.2%), while no case was observed in 835 control subjects who were long-term residents of an iodine-deficient area [81], and it was subsequently confirmed in other studies [82, 83]. Montella et al. have carried out a case-controlled study on the different oncological pathologies. They screened 495 patients with different types of cancer: 114 cases of liver cancer, 41 of multiple myeloma, 111 of non-Hodgkin's lymphomas, 130 of thyroid cancers, and 63 cases of Hodgkin's disease. The controls were 226 patients with no history of cancer. The relationship between each cancer and HCV infection was assessed by means of odds ratios (OR) and corresponding 95% confidence intervals.

Risks were greater for liver cancer (OR = 32.9, 95% CI 16.5–65.4, *P* < 0.0001), multiple myeloma (OR = 4.5, 95% CI 1.9–10.7, *P* = 0.0004), and B-cell non-Hodgkin's lymphoma (OR = 3.7, 95% CI 1.9–7.4, *P* = 0.0001). For Hodgkin's disease, there was no significant association (*P* = 0.3). An association between HCV and thyroid cancer was noted (OR = 2.8, 95% CI 1.2–6.3, *P* = 0.01) [83].

The prevalence of thyroid cancer was also investigated in a series of unselected 94 MC + HCV patients in comparison with a gender- and age-matched control group obtained from a sample of the general population (470 subjects). The prevalence of thyroid nodules was higher in control subjects than in MC + HCV patients (65.3% versus 54.8%), even though not significantly. Two patients with PTC were found in the MC + HCV series, while no case was observed among controls (*P* = 0.001). Lymphocytic infiltration was observed in the thyroid tissue in both MC + HCV patients with PTC [84]. Other studies have confirmed an association between AT and thyroid cancer [85, 86]. Accordingly, features of AT were observed more frequently in HCV patients than in controls suggesting that AT may be a predisposing condition for thyroid cancer [87]. Since about 15–30% of HCV patients may show an aggressive disease, for example, lung metastases, difficult to treat [88, 89], the finding of an increased prevalence of thyroid cancer in these patients is clinically relevant [90].

### 3.4. Immunopathogenesis of HCV Infection and AITD

Several molecular mechanisms have been suggested for the association of CHC with AT: (a) molecular mimicry or cross-reactivity which may occur between viral antigens and thyroidal antigens [91], (b) heat shock proteins expression in thyroid gland [92], and (c) abnormal expression of MHC class II molecules by thyrocytes [93].

An increased expression of IFN-*γ* and IFN-*γ* inducible chemokines [94, 95], in particular (C-X-C motif) ligand 10 chemokine (CXCL10), has been shown in hepatocytes and in lymphocytes of HCV infected patients, directly related to the degree of inflammation and to an increase in circulating levels of IFN-*γ* and CXCL10 [40, 96–103]. CXCL10 is one of chemokines with C-X-C motif. IP-10 activates specifically CXCR3 receptor that is a G protein-coupled receptor with seven transmembrane domains mainly expressed in T activated lymphocytes, natural-killer cells (NKs), macrophages, and B cells [104, 105]. Recent studies showed that CXCL10 expression in serum and/or tissue levels is increased in autoimmune organ-specific diseases [106], such as type 1 diabetes [107–109], or systemic rheumatological diseases like rheumatoid arthritis, systemic lupus erythematosus, systemic sclerosis [97, 110], sarcoidosis [111, 112], and psoriatic arthritis [113, 114].

High levels of CXCL10 are present in patients with AT, in particular in the presence of hypothyroidism, and an involvement of T-helper (Th)1 immune response in the induction of AT [97, 115–117], GD, and Graves' ophthalmopathy [97–100, 118] has been demonstrated, suggesting that intrathyroidal lymphocytes and/or thyrocytes may be the source of CXCL10 [119]. Furthermore, the presence of HCV in the thyroid of chronically infected patients has been recently shown [120, 121].

**Figure 1:**
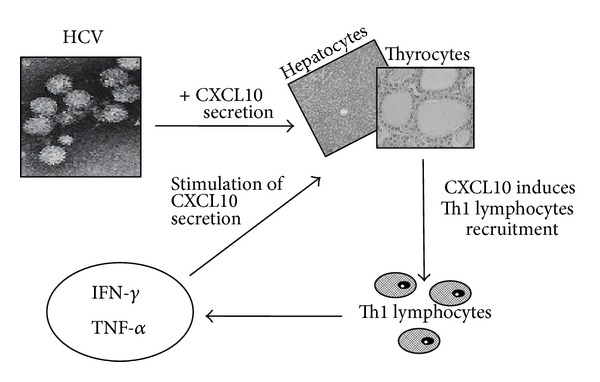
HCV thyroid infection may act by upregulating* CXCL10* gene expression and secretion in thyrocytes recruiting Th1 lymphocytes that secrete IFN-*γ* and TNF-*α*. These cytokines induce CXCL10 secretion by thyrocytes, thus perpetuating the immune cascade.

On the abovementioned bases, it has been speculated that HCV thyroid infection may act by upregulating* CXCL10* gene expression and secretion in thyrocytes recruiting Th1 lymphocytes that secrete IFN-*γ* and tumor necrosis factor- (TNF-) *α*. These cytokines induce CXCL10 secretion by thyrocytes, thus perpetuating the immune cascade, which may lead to the appearance of AITD in genetically predisposed subjects [111] (Figure 1).

Recently, the finding of high serum levels of CXCL10 but normal levels of the prototype Th2 chemokine (C-C motif) ligand 2 (CCL2) in MC + HCV patients with AT, in comparison with patients without thyroiditis, has confirmed this hypothesis. These data suggest that the Th1 CXCL10 chemokine is specifically linked to the appearance of AT in these patients [122].

Serums CXCL10 and CCL2 were assayed in 60 MC + HCV patients, in 45 patients with “MC with AT” (MC + AT), and in controls (60 without (control 1) and 45 with AT (control 2)). CXCL10 was significantly higher in control 2 than in control 1 (*P* < 0.001), in MC than in control 1, and in MC + AT than in controls 1 and 2 and MC (*P* = 0.002). A high CXCL10 level (>mean ± SD control 1; >167 pg/mL) was present in 7% of control 1, 21% of control 2, 49% of MC, and 78% of MC + AT (*P* < 0.0001). CCL2 was significantly higher in MC and in MC + AT than in control 1 or in control 2 (*P* < 0.01). A high CCL2 level (>mean ± SD control 1; >730 pg/mL) was present in 2% of control 1, 1% of control 2, 18% MC, and 21% of MC + AT (*P* < 0.0001) [122].

Among the proinflammatory cytokines, IL-1*β* and TNF-*α* were not associated with the presence of AT in MC + HCV patients, while IL-6 was modestly but significantly increased in patients with AT [5, 123–125].

On the whole, in agreement with what was observed in other autoimmune disorders [126–129], the above reported data underline the importance of the activation of the Th1 immunity in the initiation of AT in patients with MC + HCV.

## 4. Conclusion

In conclusion, the abovementioned results show a high prevalence of AITD in patients with CHC infection. The presence of a higher risk of AT in female gender, increased circulating levels of AbTPOs, and increased risk of hypothyroidism in female gender and AbTPO-positive subjects characterized the pattern of thyroid disorders observed in HCV infection. In HCV patients with thyroid cancer, thyroidectomy is required and, if appropriate, radioiodine treatment. Patients with HCV infection with AITD where nodules occurred and in fine needle aspiration biopsy without neoplastic processes do require careful observation.
